# 18p Deletion Syndrome Originating from Rare Unbalanced Whole-Arm Translocation between Chromosomes 13 and 18: A Case Report and Literature Review

**DOI:** 10.3390/children9070987

**Published:** 2022-07-01

**Authors:** Ji Young Choi, Ja Un Moon, Da Hye Yoon, Jisook Yim, Myungshin Kim, Min Ho Jung

**Affiliations:** 1 Department of Pediatrics, Yeouido St. Mary’s Hospital, College of Medicine, The Catholic University of Korea, Seoul 07345, Korea; cjy5436@hanmail.net (J.Y.C.); go--baby@hanmail.net (D.H.Y.); jmhpe@catholic.ac.kr (M.H.J.); 2 Department of Laboratory Medicine, Seoul St. Mary’s Hospital, College of Medicine, The Catholic University of Korea, Seoul 06591, Korea; newsroomw@gmail.com (J.Y.); microkim@catholic.ac.kr (M.K.)

**Keywords:** 18p deletion syndrome, monosomy 18p, unbalanced translocation, chromosomal aberration, intellectual disability, short stature, facial dysmorphism

## Abstract

18p deletion (18p-) syndrome is a rare chromosome abnormality that has a wide range of phenotypes, with short stature, intellectual disability, and facial dysmorphism being the main clinical features. Here, we report the first case in Korea of a 16-year-old male adolescent with 18p- syndrome resulting from de novo unbalanced whole-arm translocation between chromosomes 13 and 18 (45, XY, der(13;18)(q10:q10)). Three rare clinical findings were discovered that had not been reported in the previous literature; morbid obesity without other hormonal disturbances, rib cage deformity leading to the direct compression of the liver, and lumbar spondylolisthesis at the L5-S1 level. This case expands the phenotypic spectrum of 18p- syndrome and highlights the importance of considering chromosomal analysis, since this syndrome can be easily overlooked in a clinical setting, especially without distinctive symptoms of other organs, due to its nonspecific but typical features of short stature and mild intellectual disability with a mildly dysmorphic face. Moreover, since not all cases of 18p- syndrome with unbalanced translocation (13;18) show the same phenotype, multidisciplinary examinations and follow-up seem to be important to monitor evolving and developing clinical manifestations and to predict prognosis in advance associated with the specific genes of 18p breakpoint regions.

## 1. Introduction

18p deletion (18p-) syndrome, also known as monosomy 18p, is a chromosomal disorder resulting from a deletion of all or a portion of the short arm (p) of chromosome 18. Common clinical features include short stature; mild to moderate intellectual disability, especially with marked speech delay; and facial dysmorphism (e.g., a round face, ptosis, strabismus, flat nasal bridge, large protruding ears, short philtrum, and micrognathism) [1]. While most cases (about 85%) of 18p- syndrome occur from spontaneous (de novo) deletion, the remaining 15% occur due to cryptic subtelomeric deletions, ring chromosome 18, the malsegregation of familial balanced translocation, or direct familial transmission and unbalanced translocation [2,3]. Most unbalanced translocations resulting in 18p- syndrome involve the long arm of chromosome 18 and the long arm of an acrocentric chromosome such as 13, 14, 15, or especially 21 and 22, which arise either from a carrier parent or de novo [4].

To the best of our knowledge, there are only four previously published cases of unbalanced translocations occurring between chromosomes 13 and 18 (13;18) associated with 18p- syndrome [5,6,7,8], and there are none in the East Asian literature.

Herein, we report a rare case of an adolescent male diagnosed with 18p- syndrome as a consequence of de novo unbalanced whole-arm translocation between chromosomes 13 and 18.

## 2. Case Description

A 16-year-old male adolescent first visited the outpatient clinic of our ophthalmology department due to decreased visual acuity and strabismus in October 2020. Since the ophthalmologist discovered papilledema in both of his eyes through fundus photography and the ptosis on the right eyelid, the patient was transferred to our pediatric neurology department for further evaluation. He was the first child of a healthy parents and was born at full term after an uneventful prenatal period. Birth measurements including his height, body weight, and head circumference were all within a normal range of the 25th to 50th percentile. Around his first year of life, he had several episodes of epileptic generalized seizures, which were well controlled with valproic acid, and became completely seizure-free within three years without requiring anti-seizure medication. During his early childhood, since his acquisition of speech and language skills was significantly delayed, he received special support for expressive language in the pediatric rehabilitation department.

Upon clinical examination at age 16 years, he had a short stature (height < 3rd percentile, −2.1 standard deviation score) with morbid obesity (weight > 95%, body mass index ≥ 99th percentile) and an abnormally small penis with 5ml of testicular volume. His facial features included a round face, hypertelorism, mild ptosis of the right eyelid, a flat and long nose, and a short webbed neck. Neurological examination showed abnormal balance and coordination difficulty; tandem walking more than five steps was not possible; and the finger to nose test showed a marked tremor with an amplitude of less than 2 cm. He also had slurred speech and horizontal jerk nystagmus in his lateral gaze. Other examinations of muscle tone, deep tendon reflex, and vestibular function all appeared normal. Routine laboratory evaluations, including a urine metabolic screening test and blood test, were normal. Despite his short stature, micropenis, and small testicles, his growth hormones (IGF1 273 ng/mL, IGF-BP3 3740 ng/mL), gonadal hormones (LH 5.1 IU/L, FSH 8.6 IU/L, testosterone 5.5 ng/mL), as well as thyroid hormones were normal. The intelligence test showed a full-scale intelligence quotient (IQ) of 62 through the method of the Korean Wechsler Adult Intelligence Scale-IV, suggesting mild intellectual disability. However, long before the results of these tests were known, he had attended a special education school for children with intellectual disabilities. There were no specific findings from electroencephalogram and brain magnetic resonance imaging (MRI).

**Figure 1:**
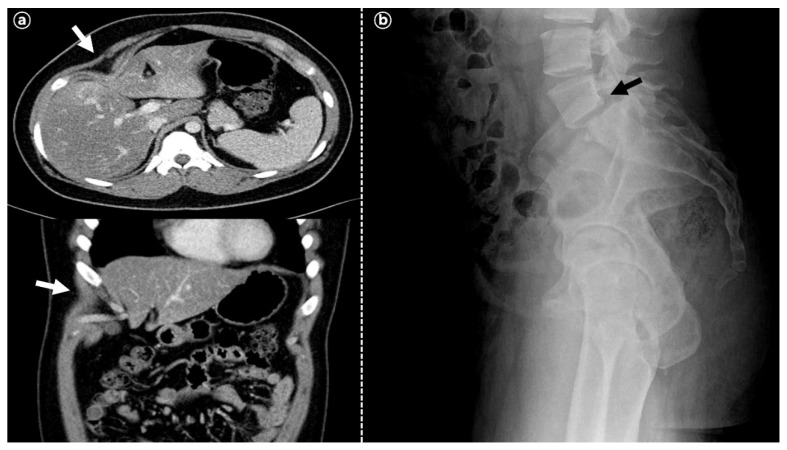
Skeletal deformities of the patient. (**a**) Axial and coronal CT scan sections showing abnormally curved right 8th rib and costal that directly compress the liver (white arrow); (**b**) sagittal lumbosacral X ray showing spondylolisthesis of L5-S1 (black arrow).

Other examinations including of the heart, were also normal, except for two unusual findings in the skeletal system. First, the right 8th rib and costal cartilage were abnormally curved, directly compressing the liver in computed tomographic (CT) findings, without a notable change in external appearance (Figure 1a). Second, the vertebral body of L5 on S1 had slipped forward out of the proper position, indicating spondylolisthesis, as found in a spine x-ray (Figure 1b).

**Figure 2:**
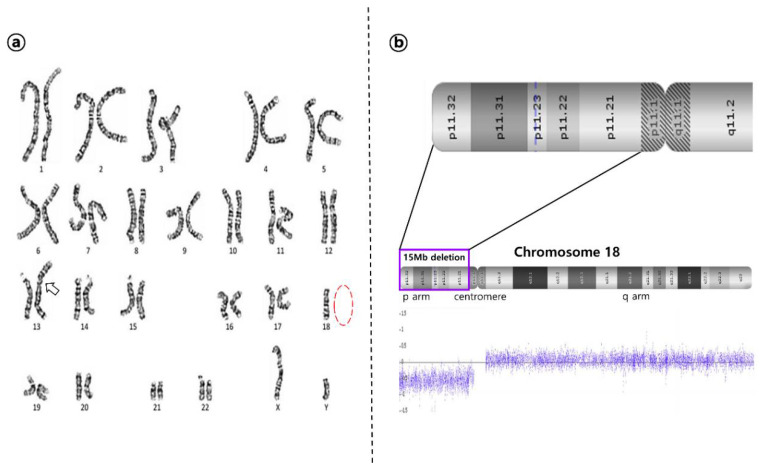
Cytogenetic results. (**a**) The karyotype of proband: 45, XY, der(13;18) (q10;q10), indicating unbalanced translocation between the long arm of one chromosome 13 and the long arm of one chromosome 18 (white arrow); (**b**) array-based comparative genomic hybridization (aCGH) of proband revealed as 15 Mb deletion in the short arm from 18p11.32 to p11.21.

A chromosomal analysis of the patent performed on peripheral blood using conventional G-banding techniques revealed an unbalanced translocation between the long arm of one chromosome 13 and the long arm of one chromosome 18, resulting in the karyotype of 45, XY, der(13;18) (q10;q10) (Figure 2a). Further genomic DNA taken from peripheral blood was analyzed using array-based comparative genomic hybridization (aCGH) with a SurePrint G3 Human CGH Microarray 8 × 60 K kit (Agilent Technologies, Santa Clara, CA, USA) in order to identify the size and location of genetic deletion/gains which often accompany chromosomal abnormalities. Control DNA (Promega Corp., Nepean, ON, Canada) was used as a reference. The patient’s result was finally revealed as arr[GRch37]18p11.32p11.21(15 Mb), indicating that chromosome 18 had a deletion in most of the short arm from 18p11.32 to p11.21 which had not been detected previously by karyotyping (Figure 2b). His parents refused permission for a cytogenetic assessment to be conducted in our hospital and instead carried it out at another hospital. Finally, their results revealed that he had a normal karyotype, indicating that a de novo translocation between the long arms of chromosome 13 and 18 had resulted in 18p- syndrome in this case.

## 3. Discussion

18p deletion syndrome, first described by de Grouchy et al., is now recognized as a well-established chromosomal disorder that occurs in approximately 1/50,000 live births, and the female to male ratio is 3:2 [1,9]. More than 300 cases have been reported in the literature since 1693, including the first case, which was published in Korea in 1981 [1]. This syndrome has a diverse range of clinical manifestations, ranging from almost asymptomatic to severe brain malformations which lead to poor prognosis. The severity of clinical phenotype tends to be correlated with the size of deletion of 18p and the loss of functional genes in critical region at 18p breakpoints [10,11,12]. In addition to the common clinical manifestations (e.g., short stature, dysmorphic face, and intellectual disability) of this syndrome, skeletal deformities including kyphoscoliosis and pectus excavatum, as well as ophthalmic complications (e.g., strabismus, ptosis, hyperopia), muscular hypotonia, and excessive tooth decay are also commonly observed [1,10,12]. Cardiac anomalies (e.g., atrial and ventricular septal defects, pulmonary stenosis), growth hormone deficiency, and neurological disorders such as movement disorders (e.g., dystonia and ataxia) and seizures have also been reported, but less frequently [12,13]. Brain malformations, the most severe phenotype of this syndrome, as part of the holoprosencephaly (HPE) spectrum, have also been reported in up to 10% of subjects with this syndrome [14]. Although children with brain malformations are more likely to have seizures, our patient developed seizures around the first year of his life without any structural abnormality, and they have been well controlled so far.

Despite the large size of the deletion found, our case showed relatively less distinctive dysmorphism and mild intellectual disability compared with other severe cases of 18p- syndrome presenting with HPE spectrum, congenital heart diseases, and severe immune deficiencies. Therefore, this case supports the severity of the phenotypic manifestation associated with functional gene loss at the 18p breakpoints [11,12,14]. Other published cases have identified an absence of specific functional genes in critical regions, causing clinical manifestations of 18p- syndrome that are consistent to some degree with our findings; these include a round face in the most distal part of 18p and short stature and seizures in 18p11.32 [1,10,15]. In addition, it is possible that the mild phenotypic abnormalities seen in our case might have resulted from the preserved segment of p11.1, whereas deletions in the centromeric region of 18p (between p11.1 and p11.21) are associated with typical facial dysmorphism and moderate to severe intellectual disabilities [12]. Moreover, the functional gene AFG3-like-AAA-ATPase-2 (*AFG3L2*) identified in 18p breakpoints has been linked to spinocerebellar ataxia type 28 with an early adolescent onset, and presents as progressive ataxia, dysarthria, nystagmus, and ptosis, as in our case [13,16]. In addition, Guanine nucleotide-binding protein-G protein (*GNAL*) gene has been related to adult onset cranio-cervical dystonia, NADH dehydrogenase ubiquinone flavoprotein 2 (*NDUFV2*) gene has been related with susceptibility to Parkinson disease, and Structural Maintenance Of Chromosomes Flexible Hinge Domain Containing 1 (*SMCHD1*) gene has been related to an increased risk of facioscapulohumeral muscular dystrophy [2,16].

Unlike other cases, three unique features were observed in the present case: severe obesity without other hormonal disturbances, an eighth rib deformity that directly compressed the liver without any abnormalities in liver function or external appearance, and the lumbosacral spondylolisthesis of L5 on S1. Of more than 300 cases of 18p- syndrome recorded, around 20 were the result of translocation between chromosome 18 and one of the acrocentric chromosomes. Nevertheless, as far as we know, only four cases of 18p- syndrome associated with translocation (13;18) have been described in the literature (Table 1). Regarding the most recent two of the four known cases, both patients were diagnosed with this syndrome at an early age owing to notable symptoms in other organs; one was discovered due to symptomatic cholelithiasis, and the other was discovered due to early-onset seizures and congenital heart disease. The other two cases were initially overlooked due to their nonspecific features such as growth retardation and learning disability, similar to our case, and were diagnosed in their late 20s.

## 4. Conclusions

In conclusion, our case expands the phenotypic spectrum of 18p- syndrome and highlights the importance of considering chromosomal analysis in cases where there is any doubt, since this syndrome can be easily overlooked in a clinical setting, especially when there are no distinctive symptoms involving other organs, due to its nonspecific but typical features of short stature and mild intellectual disability with a mildly dysmorphic face. Thus, it is important to differentiate 18p- syndrome associated with unbalanced translocation (13;18) from other neurodevelopmental disorders featuring mild intellectual disability and short stature. According to clinical phenotypes that have previously been described in adults with 18p- syndrome related to genes located at 18p11.32-11.21, as in case, new late-onset features such as dystonia, Parkinson-type movement, and muscular dystrophies might develop in our patient as he ages [2,16]. Therefore, periodic multidisciplinary examinations are necessary in order to identify expected manifestations and provide additional knowledge about the features associated with 18p- syndrome to patients and their families, which could improve their quality of life, even if only a little.

## Figures and Tables

**Table 1:** Summary of the clinical characteristics and results of 18p deletion syndrome with translocation between chromosomes 13 and 18.

Variables	Case 1	Case 2	Case 3	Case 4	Our Case
Age at diagnosis	27 years	22 years	8 years	1.5 years	16 years
Sex	Male	Male	Female	Male	Male
Cytogenetic methodology	Karyotyping	Karyotyping, FISH	Karyotyping	Karyotyping	Karyotyping, aCGH
Karyotype	45, XY, −13, 18, +t(13;18) (13qter→cen→18qter)	45, XY, der(13;18) (q10;q10)	45, XX, t(13p;18p)	45, XY, der(13;18) (q10;q10)	45, XY, der(13;18) (q10;q10)
Inheritance	de novo	de novo	NA	NA	de novo
Brain imaging	NA	NA	Small foci in frontal and parietal region ^a^	Normal	Normal
Facial dysmorphism	Posteriorly rotated ears, short neck, dental caries, café-au-lait spot	Triangular broad face, large sloping forehead, epicanthal folds, long and broad nose, large low-set ears, short neck, alopecia, disarrayed teeth, dental caries	Dolichocephaly, hypertelorism, flat nasal bridge, high arched palate, prominent forehead	Brachycephaly, hypertelorism, protruding eyes, hypodontia	Round face, hypertelorism, flat and long nose, short neck
Low birth weight	(-)	(+), 1300 g	(+), 2250 g	NA	(-)
Short stature	(+)	(+)	(+)	(+)	(+)
Language disorder	(+)	(+)	(+)	(+)	(+)
DD/ID ^b^	Borderline	Moderate	Mild	Global DD ^c^	Mild
Behavioral features	Impulse control disorder	Intermittent explosive disorder	(-)	NA	(-)
Neurological features	(-)	(-)	Seizures	Seizures	Seizures, ataxic-like movement, dysarthria
Ophthalmologic features	Strabismus	Strabismus, ptosis, nystagmus	Strabismus, microcornea	Ptosis	Strabismus, ptosis, nystagmus, pseudopapilledema
Cardiac features	NA	NA	NA	Patent ductus arteriosus, pulmonary atresia, ventricular septal defect	(-)
Endocrinological features	(-)	Acquired hypothyroidism at 40 s	(-)	NA	(-)
Skeletal features	Short left 4th metacarpal bone	Mild kyphosis, pectus carinatum	(-)	NA	8th rib cage deformity, spondylolisthesis of L5 on S1
Other features	Cutaneous basal cell carcinoma (right arm)	(-)	Everted umbilicus, gall bladder calculi	(-)	Morbid obesity, micropenis
References (year)	Moedjono SJ et al. [5] 1979	de Ravel TJ et al. [6] 2005	Nema et al. [7] 2016	Safavi et al. [8] 2019	

FISH, fluorescence in situ hybridization; aCGH, microarray-based comparative genomic hybridization; DD, developmental delay; ID, intellectual disability; NA, not applicable. ^a^ Hyperintensity in T2-weighted image from brain magnetic resonance imaging. ^b^ The severity of ID is classified by intelligence quotient (IQ) as either mild (IQ of 50–69), moderate (IQ of 35–49), severe (IQ of 20–34), or profound (IQ of <20). ^c^ Global DD is defined as significant developmental delay in two or more of the following areas: gross and fine motor skills, speech and language, cognition, and personal and social skills (≤6 years). In short, considering that most 18p deletion syndrome cases have intellectual disability with an average IQ of between 45 and 50, unbalanced translocation (13;18) associated with 18p- syndrome seems to have a mild phenotype and more favorable outcomes than other 18p- syndrome cases. Moreover, since none of these cases had an HPE spectrum, a fetal karyotype of 18p- syndrome with unbalanced translocation (13;18) resulting in fetal death has not been reported so far. However, since not all those with 18p- syndrome resulting from unbalanced translocation (13;18) have the same phenotype, other genetic factors should be considered. Moreover, considering the upper-middle socio-economic status and early support consisting of special school attendance in this case, environmental factors could have played an additional role in the patient’s favorable outcome as well [15]. Therefore, identifying the etiology of the untypical and varied clinical phenotypes of this syndrome is still challenging. Behavioral problems occurred with increased age in cases 1 and 2 and acquired hypothyroidism developed when the patient was in their 40s in case 2, indicating that clinical concern regarding not only visual problems and scoliosis but also follow-up examinations and investigations of thyroid function and adaptive behavioral skills seems to be inevitable.

## Data Availability

The data presented in this study are available upon request from the corresponding author. The data are not publicly available due to privacy restrictions.
